# High‐angular resolution diffusion imaging tractography of cerebellar pathways from newborns to young adults

**DOI:** 10.1002/brb3.589

**Published:** 2016-10-29

**Authors:** Thomas J. Re, Jacob Levman, Ashley R. Lim, Andrea Righini, Patricia Ellen Grant, Emi Takahashi

**Affiliations:** ^1^ Department of RadiologyBoston Children's HospitalHarvard Medical SchoolBostonMAUSA; ^2^ Fetal‐Neonatal Brain Imaging and Developmental Science CenterBoston Children's HospitalHarvard Medical SchoolBostonMAUSA; ^3^ Department of RadiologyUniversity of MilanMilanItaly; ^4^ Division of Newborn MedicineDepartment of MedicineBoston Children's HospitalHarvard Medical SchoolBostonMAUSA; ^5^ Department of Behavioral NeuroscienceNortheastern UniversityBostonMAUSA; ^6^ Department of Pediatric Radiology and NeuroradiologyChildren's Hospital V. BuzziMilanItaly

**Keywords:** cerebellum, MRI, pediatric, tractography

## Abstract

**Introduction:**

Many neurologic and psychiatric disorders are thought to be due to, or result in, developmental errors in neuronal cerebellar connectivity. In this connectivity analysis, we studied the developmental time‐course of cerebellar peduncle pathways in pediatric and young adult subjects.

**Methods:**

A cohort of 80 subjects, newborns to young adults, was studied on a 3T MR system with 30 diffusion‐weighted measurements with high‐angular resolution diffusion imaging (HARDI) tractography.

**Results:**

Qualitative and quantitative results were analyzed for age‐based variation. In subjects of all ages, the superior cerebellar peduncle pathway (SCP) and two distinct subpathways of the middle cerebellar peduncle (MCP), as described in previous *ex vivo* studies, were identified in vivo with this technique: pathways between the rostral pons and inferior‐lateral cerebellum (MCP *cog*), associated predominantly with higher cognitive function, and pathways between the caudal pons and superior‐medial cerebellum (MCP *mot*), associated predominantly with motor function.

**Discussion:**

Our findings showed that the inferior cerebellar peduncle pathway (ICP), involved primarily in proprioception and balance appears to have a later onset followed by more rapid development than that exhibited in other tracts. We hope that this study may provide an initial point of reference for future studies of normal and pathologic development of cerebellar connectivity.

## 1 Introduction

Noted as one of the last structures to mature, the cerebellum plays a major role in motor function, coordination, cognition, and emotion (Desmond & Fiez, 1998; Schmahmann, 1991, 1996, 2010; Schmahmann & Sherman, 1998; Schutter & van Honk, 2005). Lesions in the cerebellum have resulted in disorders of executive function (Courchesne et al., 1994; Tanaka, Harada, Arai, & Hirata, 2003), visuospatial abilities (Fabbro et al., 2004; Schmahmann & Sherman, 1998), expressive language (Fabbro et al., 2004; Molinari, Leggio, & Silveri, 1997), and affective behavior (Courchesne et al., 1994; Schmahmann, 2000), among others. Cerebellar dysfunction has also been implicated in disorders such as such autism (Courchesne et al., 1994; Fatemi et al., 2012; Penn, 2006), schizophrenia (Lungu et al., 2013; Picard, Amado, Mouchet‐Mages, Olié, & Krebs, 2008; Varambally, Venkatasubramanian, Thirthalli, Janakiramaiah, & Gangadhar, 2006), depression (Beyer & Krishnan, 2002; Leroi et al., 2002), and bipolar disorder (Beyer & Krishnan, 2002; Mills, Delbello, Adler, & Strakowski, 2005). Some of these disorders have been shown to specifically involve the cerebellar peduncle pathways (Hanaei et al., 2013; Hüttlova et al., 2014; Ojemann et al., 2013; Wang, Fan, Xu, & Wang, 2014; Wang et al., 2003, 2003, 2014) and an understanding of the development of these pathways may aid in elucidating our understanding of the development and etiology of these disorders as well as to create related diagnostic technologies.

The inferior cerebellar peduncle pathway (ICP), carries fibers primarily from the inferior olivary nuclei to the cerebellum and consist of proprioceptive and motor vestibular inputs and outputs for balance and posture maintenance (Drijkoningen et al., 2014; Ojemann et al., 2013). The middle cerebellar peduncle pathway (MCP) runs from the cerebellum to the pons and contains two distinct subpathways, which, for simplicity, will be referred to in the current work as MCP*cog* and MCP*mot* (Wang, Buckner, & Liu, 2013). MCP*cog* is predominantly associated with higher cognitive function, originates in the rostral pons and runs inferior‐laterally into the posterior cerebellum. MCP*mot* is predominantly associated with motor‐sensory function, originates in the caudal pons and runs superior‐medially toward the anterior cerebellum. Past studies have found evidence of a rostral pons to posterior cerebellum and caudal pons to anterior cerebellum connection (Schmahmann, Rosene, & Pandya, 2004; Spitzer & Karplus, 1907; Sunderland, 1940; Von Bechterew, 1885), which is further supported by evidence that points to a caudal pons anterior lobe of cerebellum sensorimotor representation (Buckner, Krienen, Castellanos, Diaz, & Yeo, 2011; Schmahmann, MacMore, & Vangel, 2009; Schmahmann & Pandya, 1997; Schmahmann & Sherman, 1998; Stoodley & Schmahmann, 2010). The superior cerebellar peduncle pathway (SCP) consists primarily of efferent output neurons traveling from the cerebellum to higher brain centers via the mid‐brain (Hüttlova et al., 2014; Mittal et al., 2014; Ojemann et al., 2013; Schmahmann et al., 2004; Takahashi, Song, Folkerth, Grant, & Schmahmann, 2013; Von Bechterew, 1885; Wang et al., 2003).

High‐angular resolution diffusion MR imaging (HARDI) tractography enables identification of complex crossing tissue coherence in the brain (Tuch et al., 2002), even in immature fetal brains (Takahashi, Folkerth, Galaburda, & Grant, 2012; Takahashi et al., 2011), which are typically more challenging to segment due to a surplus of unmyelinated fibers. This technique theoretically provides an advantage over traditional diffusion tensor imaging (DTI) in the study of intertwined and crossing fiber tracts (Frank, 2002; Tournier, Calamante, & Connelly, 2007; Tournier, Calamante, Gadian, & Connelly, 2004) which are common among cerebellar peduncular tracts. Although several techniques to assess white matter development have been developed in addition to diffusion MR tractography (Ball et al., 2013; O'Muircheartaigh et al., 2014), advantages of diffusion tractography include the detection of three‐dimensional fiber bundle pathways. Many research studies have investigated white matter pathways in adults using diffusion tractography (Cercignani, Embleton, Parker, & Bozzali, 2012; Chao et al., 2009; Jin et al., 2011; Racine et al., 2014; Thong et al., 2014; Trojsi et al., 2013; Varentsova, Zhang, & Arfanakis, 2014). However, there have been far fewer studies on the development of pathways from birth to adult ages. A few studies (Cancelliere et al., 2013; Uda et al., 2015) investigated white matter pathways from infant to adult ages looking at growth curves of pathways, but without specifically studying the cerebellar pathways and without considering laterality.

In this work, we study the cerebellar peduncle pathways in a population of pediatric and young adult subject, with normal brain MRI reports, using high‐angular resolution diffusion imaging (HARDI) (Tournier et al., 2008; Tuch et al., 2002). Our aim is to improve the knowledge of development of these pathways and to create an initial framework for future studies of normal and pathologic development of these tracts.

## 2 Materials and Methods

### 2.1 Imaging

This is a retrospective study of clinical MRI data, and the Institutional Review Board at Boston Children's Hospital deemed this an exempt project because the research is retrospective and involved existing data with no risk to patient confidentiality.

Subjects were retrospectively identified from our institution's picture archiving and communication systems (PACS), containing patient and volunteer subject imaging data for brain MRI exams performed between December 1, 2008 and June 30, 2011. The following inclusion criteria were used: (1) subject age at time of exam must have been below 30 years, (2) MRI exams must have been performed on a 3T scanner and included T1W, T2W as well as our standard diffusion‐weighted imaging sequence with 30 directions (described below), (3) MRI examinations must have been reported as normal. Exclusion criteria were as follows: (1) any reported MRI abnormality, (2) motion or other artifacts noted in the MRI report or on image inspection in consensus of two of the authors of this paper (TJR and ET). The final sample of this study consisted of 80 subjects (42 females and 38 males) ranging in age from 30 gestational weeks (GW) to 28 years (Y).

Tractography sequences consisted of 30 diffusion‐weighted measurements (b = 1,000 s/mm^2^) and five non‐diffusion‐weighted measurements (b = 0 s/mm^2^) acquired on a 3T MR system (Skyra, Siemens Medical Systems, Erlangen. Germany) with TR = 10 s; TE = 88 ms; δ = 12.0 ms; Δ = 24.2 ms; FOV = 22 × 22 cm; slice thickness = 2.0 mm; matrix size = 128 × 128, iPAT = 2.

### 2.2 Diffusion data reconstruction for tractography

*DiffusionToolkit/TrackVis* (RRID:SCR_004817; trackvis.org) was used to reconstruct and visualize tractography pathways. Tractography pathways were reconstructed using a HARDI (Q‐ball) model with a streamline algorithm and a 45° angle threshold. No threshold of fractional anisotropy (FA) was used for the fiber reconstruction as in our previous research studies (Takahashi et al., 2013).

### 2.3 Tract delineation

**Figure 1:**
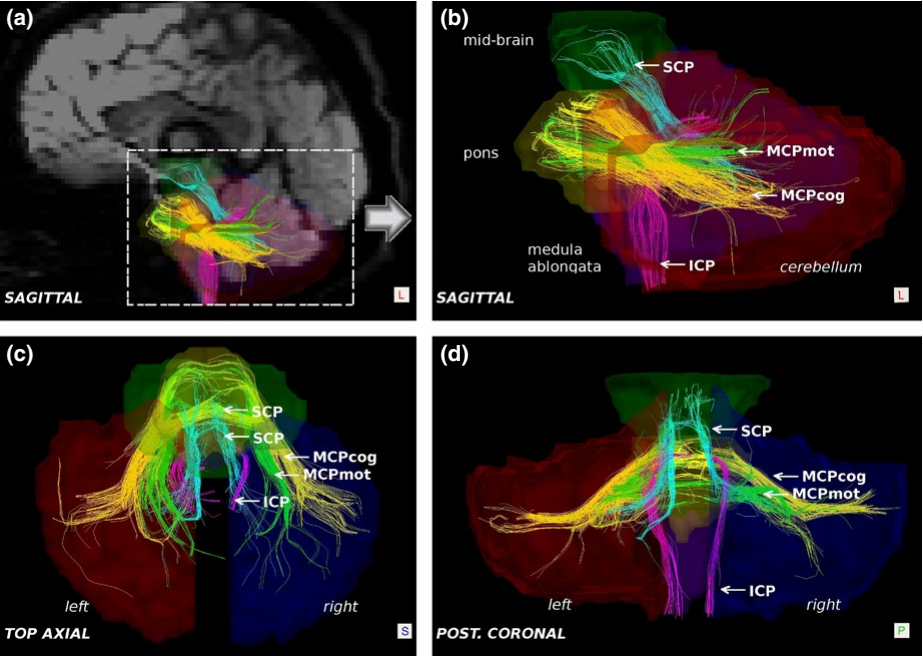
Example tractography representation of cerebellar peduncle pathways in a young adult (21Y). (a) Left sagittal whole‐brain view showing tractography results superimposed on a medial‐sagittal DWI image. Also indicated in this panel is the region of focus displayed in other panels (dashed line rectangle). (b) Left sagittal view focused on cerebellar‐brainstem region. (c) Top axial view. (d) Posterior coronal view. Anatomical structures shown in transparency as labeled. Tracts are color‐coded as: superior cerebellar peduncle pathway (SCP) in light blue, inferior cerebellar peduncle pathway (ICP) in pink, MCPcog (higher cognitive portion of MCP) in yellow, and MCPmot (motor portion of MCP) in green. See text for details

Anatomic and tractography atlases (Catani & Thiebaut de Schotten, 2008) were used to guide region‐of‐interest (ROI) placement on non‐diffusion‐weighted (b0) images and color FA maps in order to delineate the pathways of interest. ROIs were hand‐drawn on multiple slices for the mesencephalon, pons, medulla, and each cerebellar hemisphere. Figure 1 shows an example of delineated tractography pathways on one sample subject, a 21‐year‐old young adult. The four tracts studied, SCP, MCP*mot*, MCP*cog*, and ICP are labeled. The anatomic ROIs used to identify the tracts are shown in transparency and labeled in Figure 1b. The SCP pathways were defined as those with terminals in both the mesencephalon and one cerebellar hemisphere. The MCP tracts were defined as those with terminals in the pons and cerebellar hemispheres. The MCP*mot* sub‐tract was distinguished among MCP pathways via a hand‐drawn ROI, positioned interactively within the MCP, along pathways clearly originating in the caudal pons and traveling medial‐superiorly. The MCP pathways not involving this ROI (all those not identified as MCP*mot*), were considered MCP*cog* pathways. Tractography pathways corresponding to the ICP were defined as those with terminals of pathways in the medulla and the cerebellar hemisphere. Distinction was made between the left and right sides of each pathway according to which cerebellar hemisphere was involved. For each subject, a “whole‐brain” tract group was created (used for normalizing results; see below) identified as the sum of all streamlines identified within the entire intracranial brain space.

### 2.4 Quantification

Mean FA, mean ADC, number of streamlines (tract‐count) (e.g., Catani & Thiebaut de Schotten, 2008; Thiebaut de Schotten et al., 2011), mean streamline length (tract‐length) (e.g., Baker et al., 2016; Cohen et al., 2016), and total volume of all streamlines within a tract (tract‐volume) (e.g., Büchel et al., 2004; Eluvathingal, Hasan, Kramer, Fletcher, & Ewing‐Cobbs, 2007; Kulikova et al., 2014; Liu et al., 2010; Takao et al., 2011) corresponding to each cerebellar peduncle pathway (SCP, MCP*cog*, MCP*mot*, and ICP) were quantified in each subject. Left and right hemispheric values for each quantity (tract‐count, tract‐volume, mean tract‐length, mean FA, and mean ADC) in each pathway (SCP, MCP*cog*, MCP*mot*, and ICP) were compared to check for asymmetry using the Laterality Index (LI) where LI =  (ValueOnLeft – ValueOnRight)/(ValueOnLeft + ValueOnRight).

Left and right hemispheric data for each pathway (ICP, MCP*cog*, MCP*mot*, and SCP) were then combined into a single mean absolute value and also normalized according to a “whole‐brain” normalization. In *whole‐brain* normalization, each individual's quantities were compared to that of the sum of all identified brain pathways in that individual. The intent of this normalization was to account for individual variations in brain volume and tract characteristics.

### 2.5 Statistical analysis

All quantities (tract‐number, mean tract‐length, tract‐volume, mean ADC, mean FA) were plotted across age and the data were curve fitted to produce estimated “growth‐curves”. Curve fitting was performed using a default fitting procedure in Matlab (R2015a, Natick Massachusetts), with the assumption that the underlying data could be modeled by the equation: *y* = *m−ae* ^*bx*^ where *m* is the maximum data value in the samples being fitted, *a* and *b* are fitting parameters, *x* is the subject's age, and y is the resulting fitted measurement of interest. The fitting equation was selected as it was observed that the data exhibit an exponential distribution that increases with age and eventually leveling off.

Quantities were tested for left‐right asymmetry by comparing Laterality Indices to zero by way of a *t*‐test comparison with a *p*‐value <.0025 (*p *<* *.05/20) based on the Bonferroni correction of *p*‐value <.05, which was corrected for 20 comparisons.

## 3 Results

### 3.1 Superior cerebellar peduncles

**Figure 2:**
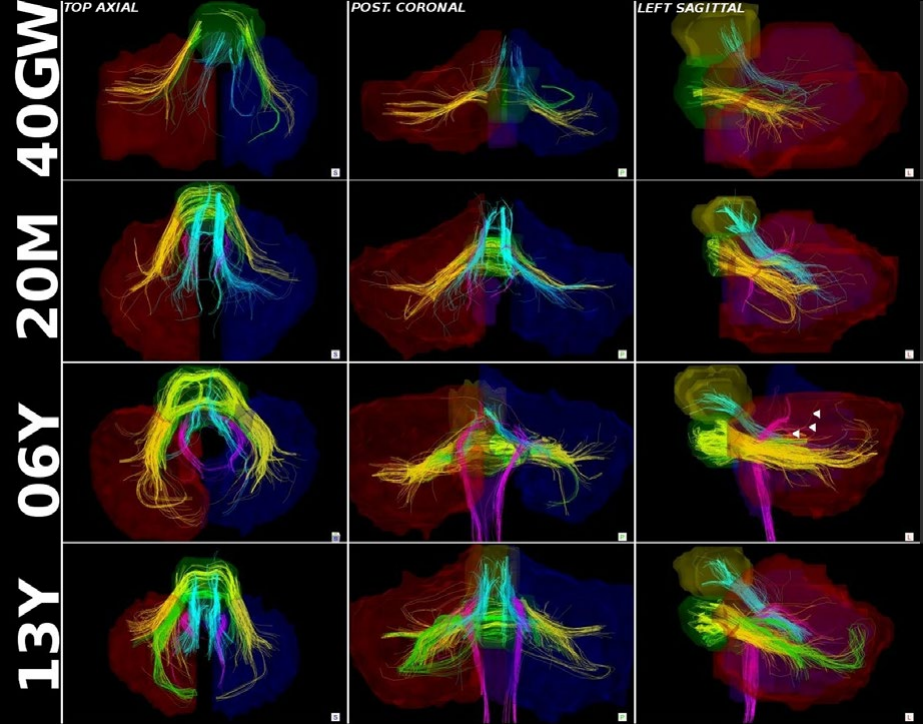
Cerebellar peduncle tracts detected with HARDI tractography in some sample subjects: a 40 gestational week (40GW) newborn, a 20 month (20M) toddler, a 6 year (6Y) child, and a 13 year (13Y) adolescent. For each, displayed from left to right: a superior‐axial (top view), posterior‐coronal, and a sagittal view are presented. Hand‐drawn ROIs used to identify tracts are shown in transparency for reference. The superior cerebellar peduncle pathway (SCP) is shown in light blue. Two subpathways of the MCP, the predominantly motor component (MCPmot, shown in green) and the predominantly higher cognitive component (MCPcog, shown in yellow) are also displayed. Inferior cerebellar peduncle pathways (ICP), shown in pink, were not generally identifiable in newborns. The ICP appears to develop rapidly between 2 and 6 months post‐term. Tractography detects more extensive branching and robustness of ICP around 6 years of age (arrowheads). Ages of subjects are shown in left margin. A set of tractography images for all subjects are available as on line supplementary material (Fig. S1)

**Figure 3:**
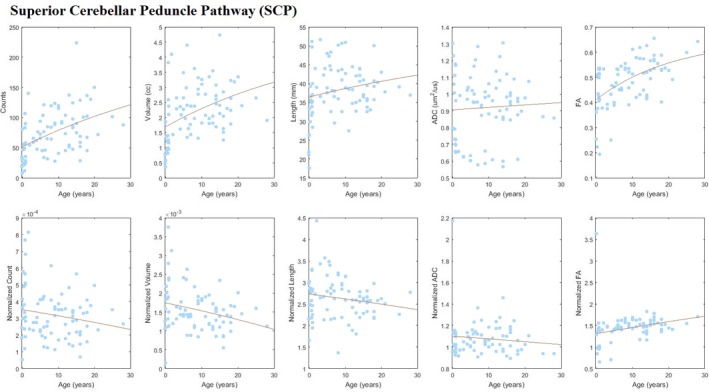
Scatter plots with fitted growth‐curves for all quantities (tract‐count, tract‐volume, mean tract‐length, mean ADC, mean FA) across ages both as absolute values and whole‐brain normalized data for superior cerebellar peduncle pathway (SCP)

Superior cerebellar peduncle pathway tracts were identified for all subjects, regardless of age (Figures 2 and S1). The fitted growth‐curves of tract‐count, tract‐volume, and mean tract‐length showed an increase in absolute value with age (Figure 3, upper panels). However, such age‐related increases appear to be lower than the overall increase across the brain as indicated by the negative slope of the curve fit for the whole‐brain normalized data (Figure 3, lower panels). SCP mean FA appeared to be increasing slightly faster than overall brain tract FA while SCP mean ADC appeared to be in‐line with that of the whole brain according to the estimated growth curves of the whole‐brain normalized values. No statistically significant left‐right asymmetry was detected for any quantitative values measured for the SCP tracts (Table 1).

**Table 1:** Asymmetry tests for cerebellar pathways. Values are *p*‐values from two‐tailed t‐test of Laterality Index (LI). Highlighted are values of statistical significance (*p *< .01). Note that only the predominantly motor component of MCP (MCPmot) shows left‐right asymmetry

Asymmetry *t*‐test results					
	Count	Volume	Length	FA	Apparent Diffusion Coefficient (ADC)
ICP	.04	.34	.46	.30	.26
MCPcog	.15	.11	.49	.22	.22
MCPmot	.00003	.0002	.07	.0009	.19
SCP	.15	.31	.05	.01	.10

ICP, inferior cerebellar peduncle pathway; SCP, superior cerebellar peduncle pathway; FA, fractional anisotropy; MCPcog, higher cognitive function component.

Values represent *t*‐test *p*‐values.

### 3.2 Middle cerebellar peduncle tracts

Sub‐tracts, MCP*cog* and MCP*mot,* were identified in all subjects regardless of age (Figures 2 and S1). The volume of MCP*cog* was significantly larger than the volume of MCP*mot* in all but three subjects (on average 9.42 ± 4.73 times larger, pairwise t‐test; *p*‐value <0.01). The three exceptions (4%), where the volume of MCP*mot* was greater than that of MCP*cog*, were at various ages (one 30W preterm; one 8M infant; and one 12Y adolescent).

**Figure 4:**
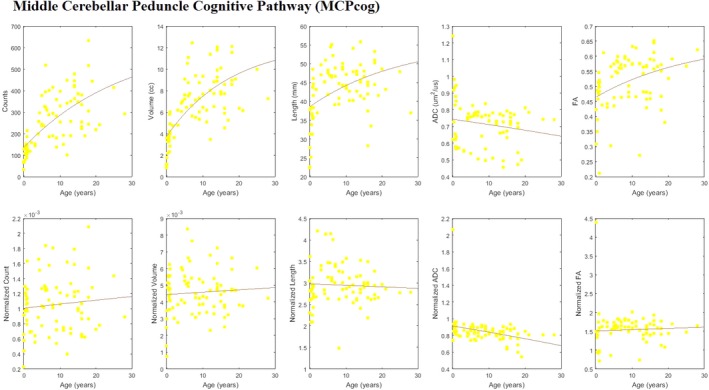
Scatter plots with fitted growth‐curves for all quantities (tract‐count, tract‐volume, mean tract‐length, mean ADC, mean FA) across ages both as absolute values (upper row) and whole‐brain normalized data (lower row) for MCP *cog*

**Figure 5:**
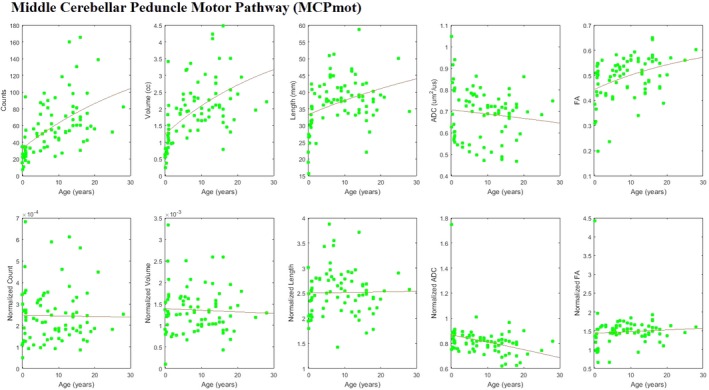
Scatter plots with fitted growth‐curves for all quantities (tract‐count, tract‐volume, mean tract‐length, mean ADC, mean FA) across ages both as absolute values and whole‐brain normalized data for MCP *mot*

For both MCP*cog* and MCP*mot*, estimated growth‐curves of tract‐count, tract‐volume, and mean tract‐length showed an increase in absolute value with age (Figures 4 and 5, upper panels). These changes were more or less in line with the changes across the whole brain as indicated by essentially flat whole‐brain normalized curves (Figures 4 and 5, lower panels).

MCP*cog* showed no statistically significant left‐right asymmetry (Table 1). MCP*mot*, however, did show statistically significant asymmetry (*p *<* *.0025) for tract‐count, tract‐volume, and mean FA (Table 1). The volumetric asymmetry was apparent on visual inspection in the majority of cases. Tract‐count and tract‐volume asymmetries were leftward in 55 of the 80 subjects (69%). Similarly, mean FA values of the MCP*mot* were higher on the left in 53 of the 80 subjects (66%). However, the direction of the tract‐count, volume, and mean FA asymmetry did not correspond in 38 of 80 (48%) of the cases.

### 3.3 Inferior cerebellar peduncle tracts

Inferior cerebellar peduncle pathways were not detected in preterm subjects (30GW and 34GW) (Fig. S1). In the three term subjects (40GW), the ICP was undetected in one of them, and only sparsely detected in the other two subjects (Fig. S1). In most subjects 2–6 months of age, the ICP pathways appear rather sparse and fragmented. These pathways appear more robust in subjects older than 6 months and appear to branch more extensively in subjects 6 years and older (Figures 2 and S1).

**Figure 6:**
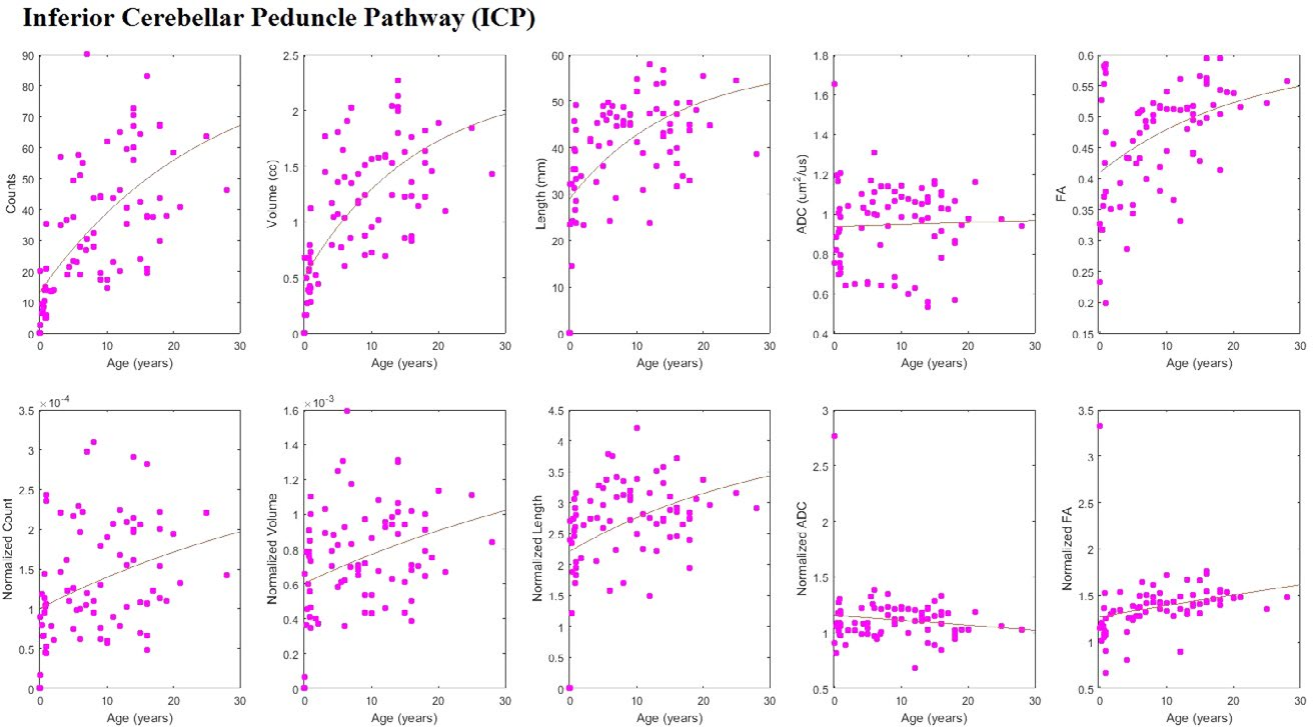
Scatter plots with fitted growth‐curves for all quantities (tract‐count, tract‐volume, mean tract‐length, mean ADC, mean FA) across ages both as absolute values and whole‐brain normalized data for inferior cerebellar peduncle pathway (ICP)

Tract‐count, tract‐volume, mean tract‐length, and mean FA all appeared to be increasing with age (Figure 6, upper panels) and at a rate faster than that of the average whole‐brain rate, as indicated by the positive slope of the curve‐fit to the whole‐brain normalized data (Figure 6, lower panels). Only ICP's mean ADC showed a substantially constant value which was in line with the whole‐brain measurements as indicated by a flat curve‐fit to normalized data.

No significant overall asymmetry was found for any quantity measured in the ICP fibers (Table 1).

### 3.4 Summary and comparison of cerebellar peduncle tracts

**Figure 7:**
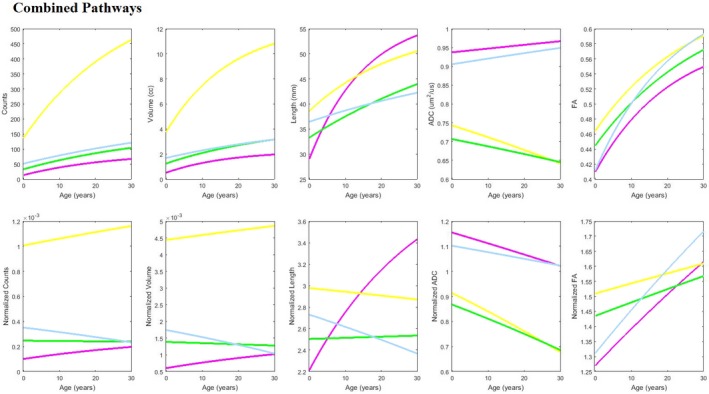
Combined fitted results for all tracts, superior cerebellar peduncle pathway (SCP), MCP *mot*, MCP *cog*, and inferior cerebellar peduncle pathway (ICP) to facilitate comparison. The data points are omitted for clarity

Combined fitted growth‐curves for all studied tracts are shown in Figure 7. Tract‐count and tract‐volume showed similar patterns in both absolute and normalized data: All studied tracts showed an increase in absolute value with age. MCP*cog* took higher values in both absolute and normalized data than those of the other studied tracts throughout the studied age range, and showed outpaced growth in absolute values. In the normalized data, MCP*cog* and ICP exhibited similar slopes with gradual increases, while SCP showed slopes with gradual decreases and MCP*mot* slopes were almost flat across ages.

Absolute values of tract‐length increased with age in all studied tracts. ICP was the shortest in newborns but became the longest in adults. Whole‐brain normalized data also supported the rapid growth of ICP, not only compared to the other studied tracts but also compared to other tracts in the brain. Normalized tract‐lengths of SCP and MCP*mot* showed similar patterns as seen in normalized tract‐count and tract‐volume: SCP showed slopes with gradual decreases and MCP*mot* slopes were almost flat across ages.

ADC results showed distinct patterns between the MCP tracts and the other tracts. Absolute ADC values of the two MCP tracts were similar to one another, taking much lower values than those of SCP and ICP throughout the studied ages. Absolute ADC values of the MCP tracts gradually decreased with age, while those of SCP and ICP slightly increased with age. Whole‐brain normalized ADC values decreased with age in all studied tracts, again exhibiting different values (low in the MCP tracts and high in the SCP and ICP tracts).

Absolute FA values greatly increased with age in all tracts, exhibiting similar growth trajectories. Normalized FA values showed a distinct pattern between the MCP tracts and the other tracts: although all four studied tracts showed increasing normalized FA values with age, the increase of the values of MCP tracts were more gradual than that of SCP and ICP.

## 4 Discussion

In this work, we studied the developmental time‐course of the cerebellar peduncle pathways using HARDI tractography in a population of subjects ranging from newborns to young adults whose brain MRI scans were reported as normal. In subjects of all ages, including in the preterm neonates, we successfully identified the SCP connecting the mesencephalon to the cerebellar hemispheres, and the two distinct subpathways of the middle cerebellar peduncle pathway (MCP): one connecting the rostral pons to the inferior‐lateral/posterior cerebellum (MCP*cog*), predominantly associated with higher cognitive function, and the other connecting the caudal pons to the superior‐medial/anterior cerebellum (MCP*mot*), predominantly associated with motor function. The inferior cerebellar peduncle pathway was not consistently identifiable with the current technique in subjects younger than 2 months of age. Absolute and whole‐brain normalized values of tract‐count, tract‐volume, tract‐length, FA, and ADC in studied tracts showed distinct growth patterns with age: absolute and whole‐brain normalized tract‐count and tract‐volume successfully dissociated between MCP*cog* and the other studied tracts, and absolute and whole‐brain normalized ADC values were found to be useful to characterize the MCP tracts and the other studied tracts. The ICP, involved primarily in proprioception and balance appears to show a later onset followed by more rapid development, especially in tract‐length, than the other pathways of the cerebellar peduncles. More precisely, ICP length appeared to go through a rapid maturation particularly during the first 6 months after birth. These findings may be in response to the infant's early extrauterine experiences with movement.

### 4.1 Superior cerebellar peduncle pathway and MCP growth patterns

Superior cerebellar peduncle pathway growth patterns of studied measurements almost always took a similar course of at least one of the other studied tracts. Absolute values of tract‐count, tract‐volume, and tract‐length of the SCP exhibited similar patterns of those from MCP*mot*. Whole‐brain normalized values of those three measures in the SCP showed a moderate decrease with age, which can be potentially useful measures to characterize SCP growth. ADC and FA values of the SCP were similar to those of ICP in both absolute and normalized values.

MCP*cog* had clearly distinguishable growth patterns in both absolute and normalized tract‐count and tract‐volume, indicating it is predominantly massive among the studied tracts. Interestingly, growth patterns of absolute/normalized ADC and normalized FA values clearly differentiated MCP*cog* and MCP*mot* from SCP and ICP. MCP*cog* and MCP*mot* exhibited lower values in absolute and normalized ADC throughout the studied ages and in early development exhibited higher normalized FA measurements but similar results in later developmental years compared to SCP and ICP. These differential growth patterns suggest these results can be useful as a reference for typical development of these tracts.

### 4.2 Inferior cerebellar peduncle pathway growth pattern

Given the nature of diffusion imaging, the absence of diffusion tractography pathways is not directly inferred to the nonexistence of axonal fibers. However, the absence of tractography pathways is likely to indicate lower mean FA due to lower myelination and lower density of axons, suggesting lower maturity of the pathways (Paus et al., 2001; Schmithorst, Wilke, Dardzinski, & Holland, 2002). Therefore, one interpretation of our findings regarding the ICP pathways (specifically, of its scarcity until 6 months post‐term) is that this particular pathway remains relatively less mature than the other pathways until it undergoes a rapid maturation during the first 6 months postpartum. Given that the ICP tends to be the thinnest tract among the cerebellar peduncles, the theory that the apparent sparsity of ICP streamlines found in the youngest subjects may be attributable to the resolution‐dependent limits of the technique cannot be excluded. However, as shown in the tractography samples of Figure 1 and of the Fig. S1, it is not always true that ICP is thinner than SCP in all subjects. Therefore, the apparent lack or sparsity of the ICP in all the subjects below 6 months of age would, in the least, reflect a later onset followed by a more rapid development of the ICP when compared to MCP and SCP. This is seen to some extent in the growth‐curves of Figure 7. The tract‐length plot demonstrates a steep increase with age from well below the value of the other tracts in the first years of life to values above the others from the teen years onward. This apparent disparity between the rapid increase in ICP mean length while the streamline count and total volume increase much more slowly, can possibly be explained by the gradual substitution of sparse disconnected fragments of the ICP detected by tractography in young subjects with more consistent full‐length streamlines in older subjects. The fragmented nature of the streamlines would likely reflect the limit of the tractography technique for properly detecting the complete but perhaps very immature neuronal pathways at this early age.

The ICP may experience a second growth surge around 6 years of life, as indicated by the increased branching of this tract in subjects at that age (Figures 2 and S1). As the ICP is related to proprioception and balance, these two periods of rapid growth may correspond to a rapid maturation of this pathway in response to the infant's early experiences with extrauterine movement and balance, and to a refinement of motor skills around 6 years of life. While these notions are still hypothetical, they merit further studies, which correlate tractography results to motor‐cognitive developmental milestones.

### 4.3 Hemispheric asymmetry

Two‐thirds of the studied subjects showed significant asymmetry of MCP*mot*, and in these cases, the asymmetry was predominantly leftward. Cerebellar functional and morphologic asymmetry has a complex pattern which is evident in both motor and higher cognitive cerebellar activities (Hu, Shen, & Zhou, 2008; Koeneke, Lutz, Wüstenberg, & Jäncke, 2004; Matsumura et al., 2004; Rosch, Ronan, Cherkas, & Gurd, 2010; Wang et al., 2013). Cerebellar motor function is primarily, although not solely, related to movements of the ipsilateral body side (Kim et al., 1993). However, the learning of new motor skills asymmetrically activate the left cerebellar motor areas regardless of the side of the body involved in the motor movements (Matsumura et al., 2004). Such leftward asymmetry of cerebellar circuits involved in motor skill learning may be reflected in cerebellar motor connectivity and provide one possible explanation for our findings.

### 4.4 Limitations of this study and future directions

This in vivo study does not provide the same level of detail of previous ex vivo studies (Takahashi, Hayashi, Schmahmann, & Ellen Grant, 2014; Takahashi et al., 2013) due to the inherit limitations of studying living subjects; however, it may provide a more practical preliminary point of reference for clinical pediatric neuroradiologists. Using the same spatial resolution under the same MR coil across subjects with different ages, smaller brains would likely be more affected by partial volume effects as compared to larger brains. For example, it would be possible for the number of tracts to be fewer in a smaller brain than in a larger brain. However, the use of a higher spatial resolution with the same number of scan repetitions reduces the signal to noise ratio, and does not guarantee the identification of a greater number of tracts in smaller brains.

As a potential issue of interdependency of our measurements, an increase in mean tract length could be associated with an increase in total tract‐count in longer streamlines which consist of a greater number of voxels and thus have a higher potential to generate a greater number of streamlines. However, as our fitted growth‐curves for these two quantities are not always superimposable, some independence most likely exists.

There were some other limitations in this study that can be addressed in the future. First, although including a large distribution of subject ages, the number of cases per age group was limited. As our findings confirm critical changes occurring within the first two years of life, a future study with a larger cohort of under 2Y subjects would be merited. Second, while our subjects had brain MRI scans which were reported as normal, they consisted in part of patients referred for MRI for clinical symptoms as well as “healthy” volunteers. The existence of imaging negative neurologic disease which could influence tractography development cannot be excluded. Third, this work combined subjects with no regard for their gender. Recent tractography studies have shown significant sex‐specific differences in white matter microstructure (Hsu et al., 2008; Kanaan et al., 2012). Future studies, aimed at establishing a normal reference standard, should consider such gender differences, as is common practice in pediatric growth curves (Barbier et al., 2013; Zong & Li, 2013). Fourth, this study's laterality analysis does not include information concerning each patient's left and right handedness which is impossible to establish for the youngest subjects. This may introduce bias as the extent to which there is an association between handedness and laterality of the brain is not fully established. Finally, for the specific goal of understanding cerebellar connectivity development, a longitudinal study might provide more insight than a cross‐sectional study.

## 5 Conclusion

This work demonstrates the usefulness of HARDI tractography for studying in vivo the normal development of the cerebellar peduncle pathways in a pediatric population. We showed that, with this technique, it is possible to identify the SCP and two subpathways of the middle cerebellar peduncle (MCP), the predominantly motor component (MCP*mot*) and the predominantly higher cognitive function component (MCP*cog*) at all ages including in premature newborns. Our results also showed that the inferior cerebellar peduncle pathway, associated with proprioception and balance, may not be readily detectable in the newborn with this technique but only after several postnatal weeks. We hope that this work contributes to our understanding of the normal development of cerebellar peduncle connectivity, and provides an initial point of reference for future tractography studies of specific cerebellar pathologies in pediatric patients (Re et al., 2015).

## Conflicts of Interest

None declared.
