# Supplementary figures and images for: High‐angular resolution diffusion imaging tractography of cerebellar pathways from newborns to young adults
Source: Brain Behav. 2016 Oct 29;7(1):e00589. doi: 10.1002/brb3.589 (PMC5256176; doi:10.1002/brb3.589)

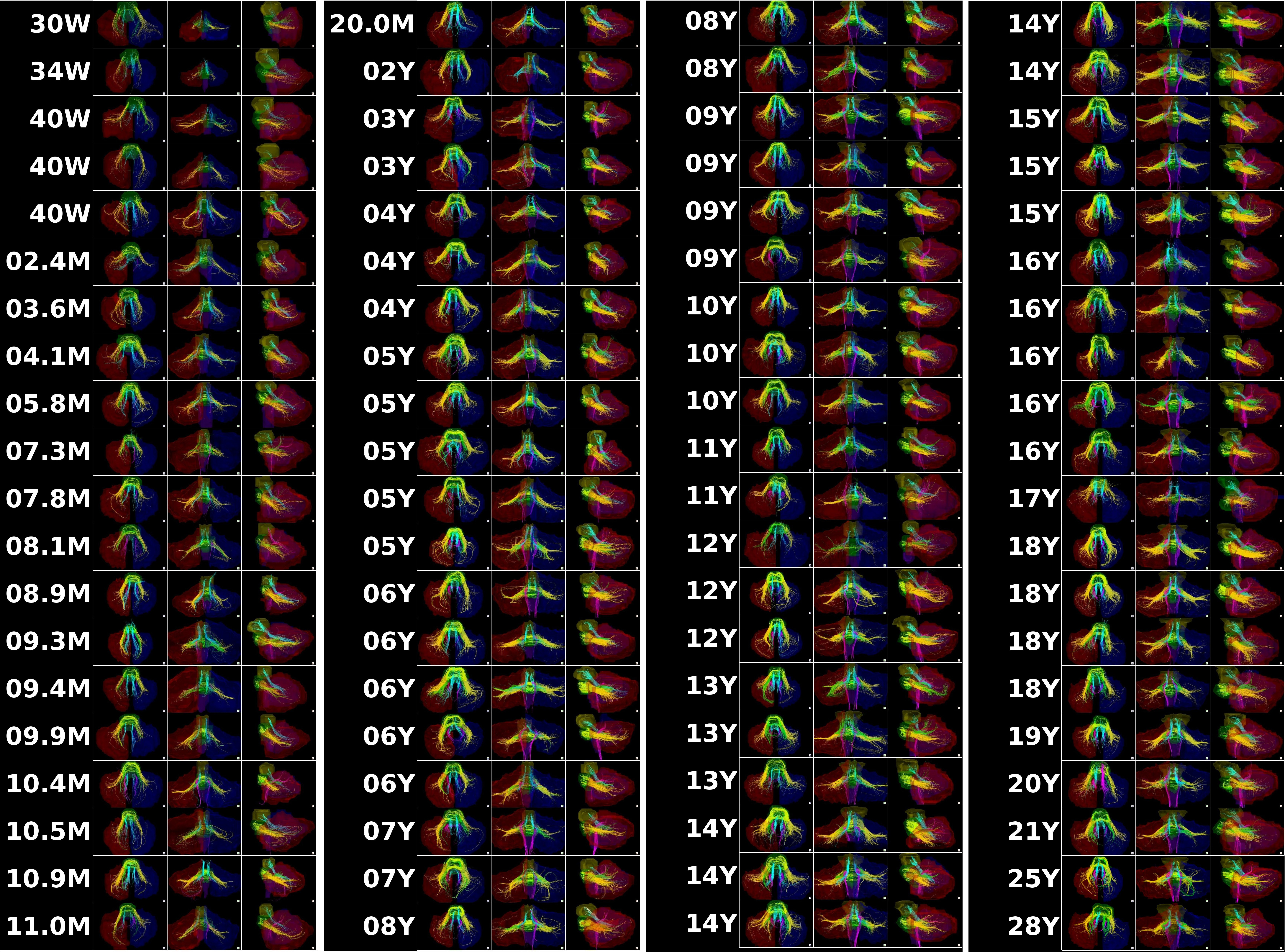

Supplement: Supplementary file 1 [file BRB3-7-e00589-s001.tif]
